# Comparison of three criteria for overweight and obesity classification in brazilian adolescents

**DOI:** 10.1186/1475-2891-12-5

**Published:** 2013-01-07

**Authors:** Andreia Pelegrini, Diego Augusto Santos Silva, Adroaldo Cezar Araujo Gaya, Edio Luiz Petroski

**Affiliations:** 1 Center for Health Sciences and Sports, Study Group in Kinanthropometry, State University of Santa Catarina, Florianópolis, Santa Catarina, Brazil; 2 Sports Center, Center for Research in Kinanthropometry and Human Performance, Federal University of Santa Catarina, Florianópolis, Santa Catarina, Brazil; 3 School of Physical Education - Department of Education, Federal University of Rio Grande do Sul, Porto Alegre, Rio Grande do Sul, Brazil

**Keywords:** Reference Standards, Body mass index, Adolescents, Nutritional status

## Abstract

**Objective:**

To describe and compare the nutritional status of adolescents using three criteria for nutritional status classification (Conde & Monteiro, International Obesity Task Force - IOTF and Word Health Organization - WHO), to analyze the correlation between these three criteria as for the overweight proportion, and to investigate whether factors associated with overweight and obesity differ among the three criteria.

**Methods:**

Demographic (gender, age, geographic area) and anthropometric (body weight, height) variables were measured in 33.728 adolescents aged 11 to 17 years. The following criteria were investigated: IOTF (2000); Conde & Monteiro (2006); and WHO (2007).

**Results:**

The overall overweight prevalence was 20.6% for the Conde & Monteiro criteria; 15.3% for the IOTF criteria and 20.1% for the WHO criteria. Both for boys and girls, the estimated overweight prevalence using the Conde & Monteiro and WHO criteria were higher than that using the IOTF criteria. Higher concordance was found between the Conde & Monteiro (2006) and WHO (2007) criteria for all age groups. Regarding associated factors, similar associations were found for the three criteria for higher BMI classification: being male, 11–12 and 13–14 years of age and living in the Midwestern, Southeastern and Southern regions of Brazil.

**Conclusion:**

The criteria for BMI classification estimate overweight prevalence in a different way, and the criteria proposed by Conde & Monteiro resulted in higher prevalence in both sexes. Higher concordance between the Conde & Monteiro and WHO criteria was found for all age groups. The groups most vulnerable to showing overweight and obesity for the three criteria for BMI classification were males, age 11–12 and 13–14 years, and living in the Midwestern, Southeastern and Southern regions of Brazil. Overweight and obesity are considered a public health problem prevalent among adolescents in Brazil, regardless of the criteria adopted.

## Background

Since the 80s, a reduction in the malnutrition prevalence among Brazilian children and adolescents has been observed [1]. However, it has been observed that the overweight and obesity prevalence in Brazilian children and adolescents (6–18 years) has tripled, jumping from 4% in the 1970s to 13% in 1997 [2]. Information from the Family Budget Survey, conducted by the Brazilian Institute of Geography and Statistics, in 2008–2009, revealed that one in three children aged five to nine years of age was overweight, and among adolescents (ten to 19 years), overweight was found to be 25.4% [3].

High overweight and obesity prevalence has been observed in developed and developing countries, and obesity is considered an important public health problem worldwide [4,5], mainly due to the close relationship between inadequate nutritional status and development of cardiovascular diseases and early mortality [6,7]. Overweight is multifactorial in origin, with important genetic [8] and environmental factors such as inadequate eating habits, for example the preference for quick meals, consisting mostly of high-calorie foods like snacks and soft drinks [9].

In this sense, the overweight and obesity diagnosis in childhood and adolescence is of great relevance to public health [10], considering that the presence of obesity in childhood and adolescence tends to persist into adulthood [11].

Despite the numerous indicators to define overweight and obesity, body mass index (BMI) has been the most common method to assess the weight status and health risks of children and adolescents [12], being widely used in epidemiological studies [13,14], due to its easy application, low operation cost and possibility of comparison with other studies. The World Health Organization [15] widely recommends the use of this anthropometric indicator for assessing the health status of people of any age.

There are several criteria for BMI classification in children and adolescents in literature [16-19]. Among them, the criteria that used samples from different countries, including Brazil, to establish cutoff points stand out, for example, Cole et al. [17] and Onis et al. [19]. Besides these criteria widely disseminated in literature, there is also the criteria of Conde & Monteiro [18], which establishes cutoff points for overweight and obesity with data exclusive of Brazilian children and adolescents. It is noteworthy that the cutoff points internationally accepted are those of CDC [16], IOTF [17] and WHO [19], while the criteria of Conde and Monteiro [18] are only valid in Brazil.

Given that overweight and obesity are increasing among young Brazilians, it is important to investigate whether the criteria used for this classification are similar in terms of overweight and obesity prevalence, as well as if the factors associated with this prevalence are the same for the different criteria used. Thus, the aim of this study was to describe and compare the nutritional status of adolescents using three criteria for nutritional status classification, and to investigate whether factors associated with overweight and obesity differ among the three different criteria.

## Methods

For this cross-sectional epidemiological study, data were collected from the “Esporte Brasil” Project (PROESP-BR) of the National Secretariat for High-Performance Sports - Ministry of Sports. This project is a permanent observatory of growth, development and nutritional status indicators of Brazilian children and adolescents of both sexes, which main objective is to define the somatomotor profile, lifestyle and motor fitness factors of children and adolescents in Brazil. More detailed information about the design and methodological aspects of the PROESP-BR project has been previously published [20].

### Population and sample

The population in this study consisted of students of both sexes aged 11 to 17 years enrolled in public and private schools from the five Brazilian regions (Northern, Northeastern, Midwestern, Southeastern and Southern). For sample selection, the PROESP-BR project was disclosed in schools and physical education teachers joined the project, assessing the students and sending data to the PROESP-BR coordination. The sample was selected by convenience in each geographical region.

In the year 2004/2005, information on about 33.728 adolescents aged 11 to 17 years (19,002 boys) integrated the PROESP-BR database. Data were collected in three states of the Midwestern region (n = 5.445), eight states of the Northeastern region (n = 4.759), five states of the Northern region (n = 3.161), four states of the Southeastern region (n = 12.614) and three states of the Southern region (n = 7.749). Of the students evaluated, 97.7% were studying in schools located in urban areas.

The Northern region of Brazil has an area of 3.659.637,9 km^2^, which represents 42.27% of the Brazilian territory, being the largest Brazilian region. The region has a population of 15.865.678 inhabitants, average human development index (HDI) of 0.764 [21]. The Northeast region of Brazil has an area of 1.558.196 km^2^, population of 53.591.197 inhabitants, and average HDI of 0.720 [21]. The Midwestern region has the country's capital, Brasilia, and has an area of 1.606.371 km^2^, population of 14.058.094 inhabitants, with a high HDI of 0.815 [21]. The Southeastern region of Brazil has the three most important cities of the country, São Paulo, Rio de Janeiro and Belo Horizonte, has the second highest HDI in Brazil, 0.824 [21] and area and population of 924.511.292 km^2^ and 80.779.802, respectively. The southern region has the highest HDI in Brazil, 0.831 [21], area of 576.409,6 km^2^ and 27.384.815 inhabitants.

Students who did not want to participate in the assessments, those who did not present the free and informed consent form signed by parents or who were absent on the day of the evaluations were considered as losses. The replacement of losses was not allowed in order not to cause a selection bias.

### Data collection procedure

Physical Education teachers who joined the project were trained and had access to instructions for the application of tests and measurements with the aid of an Internet site that included a video for standardization and better visual presentation of measurement techniques [20], prepared by members of the School of Physical Education, Federal University of Rio Grande do Sul (UFRGS), Porto Alegre, Brazil.

Throughout the fieldwork, students were informed at least one day in advance about the survey and the procedures necessary to participate. The measurements were performed preferably during physical education classes, which usually occurred in the same shift of classes of other disciplines.

Body weight was determined using a digital anthropometric scale graded from 0 to 150 kg, with accuracy of 500 g and height by means of a portable stadiometer fixed to the wall, graded from 0 to 200 cm, with precision scale of 0.2 cm [20]. BMI was calculated using the ratio between body weight in kilograms by height in squared meters.

### Outcome variable

The outcome of this study was the classification of the nutritional status obtained through the application of three criteria for BMI classification in children and adolescents. In chronological order, the first was established by the International Obesity Task Force (IOTF), published in 2000, based on data from individuals 0–25 years of age from six countries (Brazil, Britain, Hong Kong, Netherlands, Singapore and United States), collected between 1963 and 1993 [17]. The second was proposed by Conde & Monteiro in 2006 with data from the National Health and Nutrition Survey 1989, Brazil, involving subjects aged 2–19 years from all Brazilian regions [18]. The third is the WHO criteria published in 2007, which updated data from the National Center for Health Statistics (NCHS) from 1977 based on U.S. population aged 5–19 years [19].

The IOTF criteria [17] classify nutritional status into three categories (normal weight, overweight and obese). To generate the "underweight" category (thinness), data from another study carried out by the same authors were used [22]. Based on this study, participants with BMI <17 kg/m^2^ were classified as underweight (defined in the study for the adult population). As this step resulted in a few underweight adolescents in this study (<3%), they were considered as normal weight. The same procedure was adopted for the Conde & Monteiro [18] and WHO [19] criteria, which resulted in few subjects (<3%), considered as underweight. Therefore, all individuals who were underweight were kept in the database and grouped with the "normal" category. The expression overweight was adopted to define both overweight and obesity.

### Independent variables

The following were considered as independent variables and potential confounders for association analyses: sex (male, female), age in complete years and further categorized into three groups (11–12, 13–14, 15–17 years), and Brazilian geographic region (Northern, Northeastern, Midwestern, Southeastern and Southern).

### Statistical analysis

Prevalence values and confidence intervals of 95% (95%CI) were used for the nutritional status description in each of the criteria. To compare the three criteria as for the normal weight, overweight and obesity prevalence, the paired McNemar test was used; then, the test of comparison between proportions was used to identify which criteria differed according to different age groups and genders. The concordance between the diagnostic criteria was assessed by the Kappa coefficient, together with their respective 95% CI.

Finally, in order to identify factors associated with overweight and obesity according to the reference criteria, an analysis adjusted by means of Multinomial Logistic Regression was carried out, since the outcome (nutritional status) presented three categories (normal weight, overweight and obesity). Normal Weight was adopted as reference category. In the adjusted analysis, variables sex, age group and geographic region were controlled for each other. Analyses were performed in the Stata software 11.0, and in all analyses, a significance level of 5% was adopted.

### Ethical aspects

The study was approved by the Ethics Committee on Human Research of the Federal University of Santa Catarina (Protocol No. 218/08).

## Results

The sample consisted of 33.728 adolescents aged 11 to 17 years, with 19,002 males. The age group of 11–12 years comprised 15,103 individuals (53.8% of males); the age group of 13–14 years comprised 13,119 students (58.4% of males) and that of 15–17 years comprised 5,506 subjects (58.4% of males).

For boys in all age groups, the Conde & Monteiro and WHO criteria estimated higher overweight prevalence when compared to IOTF criteria. At the age group of 11–12 years, the overweight prevalence estimated by the WHO criteria was higher when that estimated by the Conde & Monteiro criteria. In the age groups of 13–14 and 15–17 years, the prevalence estimated by the Conde & Monteiro criteria was higher than that estimated by the WHO criteria (Table 1).

**Table 1:** Overweight prevalence (overweight + obesity) according to age group, in relation to three classification criteria based on body mass index among Brazilian adolescents

	**Boys**		
**Age group**	**Conde &Monteiro % (95% CI)**	**IOTF % (95% CI)**	**WHO % (95% CI)**
**Total (n = 19.002)**	19.3 (18.7-19.8)†	14.5 (14.0-15.0)	19.6 (19.0-20.1)†
**11-12 (n = 8.121)**	20.9 (20.0-21.8)†	16.4 (15.6-17.2)	23.3 (22.4-24.2)*†
**13-14 (n = 7.663)**	18.6 (17.7-19.4)†‡	13.5 (12.7-14.2)	18.1 (17.2-18.9)†
**15-17 (n = 3.218)**	16.6 (15.3-17.9)†‡	12.1 (10.9-13.3)	14.0 (12.8-15.2)†
	**Girls**		
**Total (n = 14.726)**	19.9 (19.2-20.5)†‡	14.2 (13.6-14.7)	18.0 (17.4-18.6)†
**11-12 (n = 6.982)**	24.2 (23.2-25.2)†‡	16.1 (15.2-16.9)	21.0 (20.0-21.9)†
**13-14 (n = 5.456)**	17.1 (16.1-18.1)†‡	12.6 (11.7-13.5)	16.0 (15.0-17.0)†
**15-17 (n = 2.288)**	13.3 (11.9-14.8)†	12.5 (11.2-13.9)	13.6 (12.2-15.0)†
**Total (n = 33.728)**	20.6 (20.1-21.0)†	15.3 (14.9-15.7)	20.1 (19.7-20.5)†

* Significant difference in relation to the proportion observed by the Conde & Monteiro classification criteria (p <0.05).

† Significant difference in relation to the proportion observed by the IOTF classification criteria (p <0.05).

‡ Significant difference compared to the proportion observed by the WHO classification criteria (p <0.05).

For girls (Table 1), it was observed that in all age groups, the Conde & Monteiro and WHO criteria estimated higher overweight prevalence when compared to IOTF criteria. In the age group of 11–12, 13–14 years and considering the entire sample, the prevalence estimated by the Conde & Monteiro criteria was higher than that estimated by the WHO criteria.

Table 2 shows the concordance between the three criteria for overweight diagnosis. In males, it was observed that at the age of 11–12 years, the concordance between the Conde & Monteiro and IOTF criteria was higher than that obtained between IOTF and WHO and WHO and Conde & Monteiro criteria. At the age of 13–14 years and considering all age groups, the concordance obtained between Conde & Monteiro and IOTF and WHO and Conde & Monteiro criteria was higher than that obtained between IOTF and WHO.

**Table 2:** Kappa coefficient and confidence intervals of 95% (in parentheses) for overweight diagnosis (overweight + obesity) according to three criteria for BMI classification among Brazilian adolescents

**Age group (years)**	**Sex**	**Conde & Monteiro*vs*IOTF**	**IOTF*vs*WHO**	**WHO*vs*Conde and Monteiro**
11-12	Male	0.84 (0.83-0.86)^†‡^	0.62 (0.60-0.64)	0.75 (0.74-0.77)^†^
	Female	0.67 (0.65-0.69)	0.72 (0.68-0.74)	0.89 (0.88-0.90)*^†^
	All	0.76 (0.75-0.77)^†^	0.66 (0.64-0.67)	0.82 (0.81-0.83)*^†^
13-14	Male	0.81 (0.79-0.83)^†^	0.71 (0.69-0.73)	0.85 (0.83-0.86)^†^
	Female	0.78 (0.76-0.81)	0.78 (0.75-0.80)	0.93 (0.91-0.94)*^†^
	All	0.80 (0.78-0.81)^†^	0.74 (0.72-0.76)	0.88 (0.87-0.89)^*†^
15-17	Male	0.80 (0.77-0.83)	0.82 (0.78-0.84)	0.83 (0.80-0.85)
	Female	0.94 (0.92-0.96)^†^	0.88 (0.85-0.91)	0.93 (0.91-0.95)
	All	0.86 (0.84-0.88)	0.84 (0.82-0.86)	0.87 (0.85-0.89)
All	Male	0.82 (0.81-0.83)^†^	0.68 (0.67-0.69)	0.80 (0.79-0.81)^†^
	Female	0.74 (0.73-0.75)	0.76 (0.75-0.77)	0.91 (0.90-0.92)*^†^
	All	0.79 (0.78-0.80)^†^	0.72 (0.71-0.72)	0.85 (0.84-0.86)*^†^

* Concordance greater than the comparison between Conde & Monteiro vs IOTF (for same sex and age group).

† Concordance greater than the comparison between IOTF vs WHO (for same sex and age group).

‡ Concordance greater than the comparison between WHO vs. Conde & Monteiro (for same sex and age group).

In females, it was observed that at the age of 11–12, 13–14 years and considering all age groups, the correlation between WHO and Conde & Monteiro criteria was greater than the other criteria (Table 2).

Table 3 shows the association between overweight and obesity and demographic variables according to the Conde & Monteiro classification criteria. The likelihood of adolescents of being overweight was higher among male adolescents, 11–12 and 13–14 years and living in the Midwestern and Southern regions of Brazil.

**Table 3:** Crude and adjusted multinomial logistic regression analysis (reference category = normal weight) for overweight and obesity according to criteria proposed by Conde & Monteiro for Brazilian adolescents

		**Conde & Monteiro**				
	**Overweight**			**Obesity**		
	**%**	**OR (95% CI)**	OR* (95% CI)	**%**	**OR (95% CI)**	OR* (95% CI)
**Sex**						
Male	16.6	**1.06 (1.00-1.12)**	**1.10 (1.04,1.17)**	2.6	0.61 (0.54-0.69)	0.65 (0.57, 0.73)
Female	15.6	1	1	4.3	1	1
**Age group (years)**						
11-12	18.2	**1.53 (1.40-1.67)**	**1.81 (1.65, 1.98)**	4.2	**2.03 (1.67-2.47)**	**2.48 (2.03, 3.03)**
13-14	15.2	**1.22 (1.11-1.33)**	**1.39 (1.26, 1.52)**	2.8	**1.26 (1.02-1.54)**	**1.51 (1.22, 1.86)**
15-17	13.0	1	1	2.3	1	1
**Geographic Region**						
Northeastern	13.5	0.94 (0.82-1.07)	0.94 (0.83, 1.07)	2.6	1.17 (0.88-1.57)	1.19 (0.89, 1.59)
Midwestern	16.1	**1.16 (1.03-1.31)**	**1.17 (1.04, 1.32)**	2.8	**1.30 (1.00-1.70)**	**1.33 (1.02, 1.75)**
Southeastern	15.0	1.08 (0.97-1.19)	1.08 (0.98, 1.20)	3.4	**1.58 (1.25-2.00)**	**1.60 (1.27, 2.03)**
Southern	20.5	**1.60 (1.44-1.78)**	**1.80 (1.61, 2.00)**	4.7	**2.38 (1.87-3.02)**	**2.74 (2.15, 3.50)**
Northern	14.3	1	1	2.2	1	1

* Odds ratio adjusted for all variables.

Note: Bold - variables with p-value <0.05 in the Wald test. *OR* odds ratio, *CI* confidence interval.

Regarding obesity, association was found between age group and geographic region. The likelihood of adolescents of being obese when compared to normal weight was higher in those aged 11–12 and 13–14 years and living in the Midwestern, Southeastern and Southern regions of Brazil (Table 3). Furthermore, it was found that boys were less likely to be obese compared to girls.

Table 4 shows the association between overweight and obesity and demographic variables according to the IOFT classification criteria. The results show association between age group and geographic region and overweight. The likelihood of adolescents of being overweight was higher among adolescents aged 11–12 and 13–14 years and living in the Midwestern, Southeastern and Southern regions of Brazil.

**Table 4:** Crude and adjusted multinomial logistic regression analysis (reference category = normal weight) for overweight and obesity according to criteria proposed by IOTF for Brazilian adolescents

		**IOTF**				
	**Overweight**			**Obesity**		
	**%**	**OR (95% CI)**	OR* (95% CI)	**%**	**OR (95% CI)**	OR* (95% CI)
**Sex**						
Male	11.7	0.98 (0.92-1.05)	1.02 (0.96-1.09)	2.8	**1.23 (1.07-1.42)**	**1.30 (1.14, 1.50)**
Female	12.0	1	1	2.3	1	1
**Age group (years)**						
11-12	13.2	**1.33 (1.21-1.47)**	**1.55 (1.40, 1.72)**	3.1	**1.73 (1.40-2.15)**	**2.17 (1.74, 2.71)**
13-14	10.8	**1.05 (0.95-1.16)**	**1.19 (1.07, 1.32)**	2.3	**1.27 (1.01-1.60)**	**1.51 (1.20, 1.90)**
15-17	10.4	1	1	1.9	1	1
**Geographic Region**						
Northeastern	9.8	1.06 (0.77-1.47)	1.06 (0.91, 1.24)	2.0	1.07 (0.77-1.47)	1.05 (0.76, 1.46)
Midwestern	11.4	**1.27 (1.11-1.45)**	**1.28 (1.11, 1.46)**	2.3	1.26 (0.94-1.68)	1.26 (0.94, 1.69)
Southeastern	11.3	**1.26 (1.11-1.42)**	**1.26 (1.12, 1.43)**	2.5	**1.39 (1.08-1.81)**	**1.40 (1.08, 1.81)**
Southern	15.3	**1.81 (1.60-2.05)**	**1.96 (1.73, 2.23)**	3.6	**2.15 (1.65-2.79)**	**2.49 (1.91, 3.24)**
Northern	9.3	1	1	1.9	1	1

* Odds ratio adjusted for all variables.

Note: Bold - variables with p-value <0.05 in the Wald test. *OR* odds ratio, *CI* confidence interval.

Regarding obesity, association was found between age group and geographic region. The likelihood of adolescents of being obese when compared to normal weight was higher in those aged 11–12 and 13–14 years and living in the Midwestern, Southeastern and Southern regions of Brazil (Table 4).

Table 5 shows the association between overweight and obesity and demographic variables according to the WHO classification criteria. The results show association between age group and geographic region with overweight. The likelihood of adolescents of being overweight was higher among adolescents aged 11–12 and 13–14 years and living in the Midwestern, Southeastern and Southern regions of Brazil.

**Table 5:** Crude and adjusted multinomial logistic regression analysis (reference category = normal weight) for overweight and obesity according to criteria proposed by WHO for Brazilian adolescents

		**WHO**				
	**Overweight**			**Obesity**		
	**%**	**OR (95% CI)**	OR* (95% CI)	**%**	**OR (95% CI)**	OR* (95% CI)
**Sex**						
Male	12.5	0.99 (0.93-1.06)	1.04 (0.97, 1.11)	7.2	**1.38 (1.26-1.52)**	**1.48 (1.35, 1.62)**
Female	12.7	1	1	5.3	1	1
**Age group (years)**						
11-12	14.0	**1.58 (1.43-1.75)**	**1.84 (1.66, 2.04)**	8.2	**2.28 (1.96-2.64)**	**2.81 (2.42, 3.27)**
13-14	12.1	**1.28 (1.15-1.42)**	**1.45 (1.30, 1.61)**	5.2	**1.35(1.15-1.57)**	**1.58 (1.34, 1.85)**
15-17	9.8	1	1	4.0	1	1
**Geographic Region**						
Northeastern	10.4	0.94 (0.82-1.09)	0.94 (0.81, 1.08)	5.5	1.24 (1.01-1.52)	1.22 (1.00, 1.50)
Midwestern	12.2	**1.14 (1.00-1.30)**	**1.16 (1.01, 1.31)**	6.0	**1.39 (1.15-1.67)**	**1.39 (1.15, 1.69)**
Southeastern	12.0	**1.12 (1.00-1.25)**	**1.13 (1.01, 1.26)**	6.2	**1.43 (1.20-1.69)**	**1.43 (1.21, 1.70)**
Southern	15.7	**1.57 (1.40-1.77)**	**1.77 (1.57, 1.99)**	8.2	**2.02 (1.70-2.40)**	**2.47 (2.07, 2.94)**
Northern	11.1	1	1	4.5	1	1

* Odds ratio adjusted for all variables.

Note: Bold - variables with p-value <0.05 in the Wald test. *OR* odds ratio, *CI* confidence interval.

Regarding obesity, association was found between sex, age group and geographic region. The likelihood of adolescents of being obese when compared to normal weight was higher in males, 11–12 and 13–14 years and living in the Midwestern, Southeastern and Southern regions of Brazil (Table 5).

## Discussion

This is the first study carried out with adolescents from all geographic regions of Brazil, which has characteristics of a country with continental dimensions, which different ethnicities, customs and traditions. Furthermore, this study evaluated if the factors associated (sex, age and geographic region) with overweight and obesity are the same for the different classification criteria used. It is noteworthy that the three criteria used have in common the fact of using arbitrary cutoff points and adjusting BMI values that characterize underweight, overweight and obesity in adults for the population of children and adolescents [23].

The three references used in this study showed marked differences between each other. Both for boys and girls, increased overweight prevalence was observed by the Brazilian reference of Conde & Monteiro [18], followed by WHO [19] and IOTF [17].

Some studies carried out in Brazil [24,25] and elsewhere [26,27] also compared different criteria for nutritional status classification in children and adolescents. Most of these studies demonstrated that the criteria for nutritional status classification differ, but a study conducted in adolescents of Florianópolis, Santa Catarina, Brazil, Farias Jr et al. [24] found no significant differences in the overweight prevalence determined by the different BMI classification criteria, except for male adolescents, in which the BMI values recommended by Conde & Monteiro [18] showed higher prevalences compared to the other criteria.

Contrasting these findings, a study carried out in Rio Grande, Rio Grande do Sul, Brazil [28] with students aged 10 to 15 years revealed that the overweight prevalence was significantly higher using the Conde & Monteiro [18] criteria than using IOTF [17]. In Canadian preschoolers [27], the overweight prevalence using the WHO criteria was higher than that using the IOTF and the Centers for Disease Control and Prevention (CDC) criteria [29].

One possible explanation for the higher overweight prevalence found using the criteria proposed by Conde & Monteiro [18] is due to the fact that the critical BMI values are lower than those of the other criteria, as already observed by Farias Jr et al. [24]. Moreover, it could be speculated that unlike criteria of IOTF [17] and WHO [19], the cutoff points of Conde and Monteiro [18] were defined with the specific population of Brazil. Thus, one might think that the prevalence detected by the criteria proposed by Conde and Monteiro [18] may represent the real problem of the high overweight and obesity prevalence in Brazilian adolescents.

When the Kappa test was applied, high concordance between criteria of Conde & Monteiro [18] and WHO [19] was found for all age groups and for the data set. Moreover, strong concordance (kappa> 0.75) was observed for the most overweight classifications analyzed. These results corroborate the findings of Dumith and Farias Jr [28], who also found that the concordance between criteria of Conde & Monteiro and WHO was higher than the concordance obtained between the other criteria.

Regarding associated factors, similar associations were found for the three criteria for BMI classification examined: being male, age 11–12 and 13–14 years, living in the Midwestern, Southeastern and Southern regions of Brazil had greater chances of showing overweight. A study conducted among adolescents in southern Brazil revealed that the direction and magnitude of the association measures were similar between criteria for BMI classification [28]. Corroborating these findings, Abrantes et al. [30] also observed higher overweight prevalence among adolescents in southeastern Brazil when compared to those of northeastern Brazil.

The results of this study make some hypotheses to be raised: the lower overweight prevalence in girls may be related to a greater concern with body image [31]. The decline in overweight and obesity with increasing age is expected, since moderate excess weight might be compensated by growth, and may even represent a positive aspect of the obesity treatment [32]; however, excess weight in adolescence should not be underestimated, since there is an increased risk of persisting into adulthood [33]. It is assumed that differences between regions are related to different socioeconomic development between regions [21], resulting in a greater prevalence in the most developed regions. The higher overweight prevalences found in the Midwestern, Southeastern and Southern regions can be explained due to the difference socioeconomic development between regions, resulting in a greater prevalence in more developed regions, where there are better conditions of purchase and choice of food [1]. Nevertheless, there is an increasing trend in the prevalence of overweight in the North and Northeast and the lower socioeconomic levels [1].

The present study has the following limitations: 1) the sample selection was performed only in schools that joined the PROESP project, and thereby the participation of more adolescents with obesity characteristic was limited, 2) data are restricted to adolescent students who were in school during the period of data collection and could not be extrapolated to adolescents who were not enrolled in schools.

## Conclusions

The findings of this study indicate that the criteria for BMI classification estimate overweight prevalence in a different way, and the criteria proposed by C & M resulted in higher prevalence in both sexes. Higher concordance between the Conde & Monteiro and WHO criteria was found for all age groups. The groups most vulnerable to showing overweight and obesity for the three criteria for BMI classification were males, age 11–12 and 13–14 years, and living in the Midwestern, Southeastern and Southern regions of Brazil.

## Competing interests

The authors declare that they have no competing interests.

## Authors’ contributions

AP contributed to statistical analysis, data interpretation, and manuscript writing. DASS contributed to statistical analysis, data interpretation, and manuscript writing. ACAG contributed to the study design and the data acquisition. ELP contributed to revise it critically for important intellectual content. All authors read and approved the final manuscript.
